# Neoadjuvant chemotherapy followed by cytoreductive surgery and hyperthermic intraperitoneal chemotherapy for colorectal cancer: a feasibility and safety study

**DOI:** 10.1186/s12957-018-1554-8

**Published:** 2019-01-11

**Authors:** M. Leimkühler, P. H. J. Hemmer, A. K. L. Reyners, D. J. A. de Groot, R. J. van Ginkel, L. B. Been, G. H. de Bock, B. L. van Leeuwen

**Affiliations:** 1 Department of Surgery, University of Groningen, University Medical Center Groningen, Hanzeplein 1, 9713GZ, Groningen, The Netherlands; 2 Department of Medical Oncology, University of Groningen, University Medical Center Groningen, Hanzeplein 1, 9713GZ, Groningen, The Netherlands; 3 Department of Epidemiology, University of Groningen, University Medical Center Groningen, Hanzeplein 1, 9713GZ, Groningen, The Netherlands

**Keywords:** Neoadjuvant chemotherapy, HIPEC, Colorectal cancer, Peritoneal carcinomatosis

## Abstract

**Background:**

Standard treatment for colorectal peritoneal carcinomatosis typically involves cytoreductive surgery, hyperthermic intraperitoneal chemotherapy (HIPEC), and if possible, postoperative adjuvant chemotherapy. However, a substantial percentage of patients never receive adjuvant chemotherapy because of postoperative complications. Neoadjuvant chemotherapy could be beneficial in this setting, so we assessed its feasibility and safety when used before cytoreductive surgery and HIPEC.

**Methods:**

In this non-randomized, single-center, observational feasibility study, patients were scheduled to receive six cycles of capecitabine and oxaliplatin before cytoreductive surgery and HIPEC. Computed tomography was performed after the third and sixth chemotherapy cycles to evaluate tumor response, and patients underwent cytoreductive surgery and HIPEC if there were no pulmonary and/or hepatic metastases. Postoperative complications, graded according to the Clavien–Dindo classification, were compared with those of a historic control group that received postoperative adjuvant chemotherapy.

**Results:**

Of the 14 patients included in the study, 4 and 3 had to terminate neoadjuvant chemotherapy early because of toxicity and tumor progression, respectively. Cytoreductive surgery and HIPEC were performed in eight patients, and the timing and severity of complications were comparable to those of patients in the historic control group treated without neoadjuvant chemotherapy.

**Conclusion:**

Patients with peritoneal metastases due to colorectal carcinoma can be treated safely with neoadjuvant chemotherapy before definitive therapy with cytoreductive surgery and HIPEC.

**Trial registration number:**

NTR 3905, registered on 20th march, 2013, http://www.trialregister.nl/trialreg/admin/rctview.asp?TC=3905

## Background

Peritoneal carcinomatosis is common in patients with colorectal cancer, occurring in 5–10% of those presenting with synchronous peritoneal metastases and 20–50% of those presenting with metachronous peritoneal metastases [1]. If left untreated, the median patient survival of this group is 3–9 months [2]. Therefore, peritoneal carcinomatosis has long been considered an incurable disease [3], with palliative treatment extending median progression-free survival to just 6–12 months [4–6]. However, recent developments in systemic treatment have increased the 2-year survival of patients from approximately 0% in 2000 to 10–16% in 2014 [6–8].

In recent years, many institutions started treating patients with cytoreductive surgery (CRS) and hyperthermia intraperitoneal chemotherapy (HIPEC). A 5-year survival between 33 and 58% has been described in patients with colorectal carcinoma undergoing CRS plus HIPEC [9–11]. Because of this survival benefit, CRS plus HIPEC is considered the treatment of choice for patients with low-volume, low-grade peritoneal disease without systemic dissemination [3]. Since 2006, systemic adjuvant chemotherapy has been the logical treatment for preventing hematogenous and lymphogenous spread. However, the high postoperative complication rate following CRS and HIPEC, which has ranged from 23 to 66% [2, 7, 12–15], can leave many patients ineligible for adjuvant therapy, and no studies have shown evidence of survival benefit for this approach in patients with peritoneal carcinomatosis. Nevertheless, it has also been shown that neoadjuvant chemotherapy confers survival benefits in other tumors [16–19], and on this basis, we thought that it could be used before CRS and HIPEC in patients with colorectal cancer. In this way, it might even help to reduce tumor load and lessen the extent of surgery. A possible disadvantage, however, might be that neoadjuvant chemotherapy could negatively affect the postoperative complication rate. At the time of writing, we could identify no prospective data on the efficacy of neoadjuvant systemic treatment for this indication.

We conducted this feasibility study as a prequel to a larger prospective multicenter study of the efficacy of neoadjuvant systemic treatment in patients with peritoneal carcinomatosis due to colorectal cancer. The primary aim was to determine whether neoadjuvant chemotherapy influenced postoperative complication and mortality rates after CRS and HIPEC.

## Methods

We conducted a non-randomized, single-center, observational feasibility study from April 2013 to July 2015 at the University Medical Center Groningen (UMCG), which is a referral center for HIPEC, serving 1.6 million inhabitants. A historical control cohort of patients treated with standard CRS and HIPEC, but without neoadjuvant chemotherapy, served as a reference population (20). The study was conducted in accordance with the Declaration of Helsinki and approved by the Medical Ethics Committee of the UMCG and was registered in the Dutch trial registry (NTR 3905).

### Patients

Patients were recruited from the UMCG if they had peritoneal carcinomatosis of colorectal origin diagnosed by computed tomography (CT), diagnostic laparoscopy, or laparotomy. All diagnoses were confirmed by pathological examination. The following eligibility criteria were applied: World Health Organization (WHO) performance score of 0 or 1, adequate bone marrow function (platelets > 100 × 10^9^, neutrophils > 1.5 × 10^9^), and adequate renal function (creatinine clearance > 50 mL/min).

The main exclusion criterion in the experimental group was prior treatment with adjuvant systemic chemotherapy within 12 months of the study. Patients were also excluded if they had a history of other malignancies (except basal cell carcinoma) or advanced liver disease (bilirubin > 34 μmol/L and/or an international normalized ratio > 1.7). Finally, we excluded patients with liver and/or extra-abdominal metastases or neurotoxicity above grade 1 according to The Common Terminology Criteria for Adverse Events (Version 4.0) [20]. All included patients of the experimental group provided a written informed consent.

The historic reference population consisted of patients with peritoneal carcinomatosis of colorectal origin who were treated at the UMCG between 2006 and 2015 [21]. This reference population included 88 patients who received standard treatment, consisting of CRS and HIPEC, followed by adjuvant chemotherapy. We only included patients with a peritoneal cancer index (PCI) less than 20, because only this group has been shown to benefit from HIPEC. Dutch law does not require informed consent to use anonymized patient data for treatment evaluation.

### Standard treatment

Standard treatment involves CRS plus HIPEC followed by cytoreductive surgery [22]. During surgery, all macroscopic lesions were removed, before intraperitoneal mitomycin C was given at a dose of 35 mg/m^2^ over 90 min. This chemotherapy was administered in three doses, with half given at the start of perfusion, one-quarter after 30 min, and one-quarter after 60 min; thereafter, perfusion continued for 30 min [23].

### Experimental treatment

The experimental protocol involved giving neoadjuvant chemotherapy before CRS and HIPEC. Patients enrolled to this protocol were scheduled to receive six cycles of capecitabine (1000 mg/m^2^) and oxaliplatin (130 mg/m^2^) (i.e., CAPOX) every 3 weeks. Oxaliplatin was given intravenously on the first day of each cycle and capecitabine was taken orally for 14 days in 21-day cycles. Before CRS and HIPEC were performed, patients were given a 4-week recovery period after the last cycle of chemotherapy. If the patient had not recovered satisfactorily by this time, recovery could be extended to a maximum of 10 weeks. To be eligible for surgery, patients were not allowed to have leucopenia or thrombocytopenia, and were required to have a WHO performance score of 2 or less. The CRS plus HIPEC procedure followed the protocol described under standard treatment.

CT scans were performed to provide a baseline assessment before systemic treatment, and tumor response was re-evaluated after three and six treatment cycles. These evaluations were done by a radiologist, according to the Response Evaluation Criteria in Solid Tumors (RECIST) criteria [24]. If tumor progression was seen on the follow-up CT, without extra-abdominal or liver metastases, patients went directly for CRS and HIPEC. If the tumor was non-resectable or if extra-abdominal or liver metastases were present, palliative surgery and/or palliative systemic treatment were offered.

### Data collection

Data were recorded for the following variables: primary diagnosis, age, length of hospital stay, PCI, blood loss during surgery, duration of surgery, complications during systemic chemotherapy, complications in the 30 days following CRS and HIPEC, whether the full chemotherapy cycle was completed, disease progression, and survival. Surgical complications were reported according to the Clavien–Dindo classification [25]. Complications were given by system, as follows: cardiovascular complications included arrhythmias and deep vein thrombosis; pulmonary complications included pneumonia, atelectasis, pleural effusion, and pulmonary embolism; gastrointestinal complications included gastroparesis, excessive pain, and gastric retention; mental complications included delirium and excessive fear; and neurological complications included neuropathy and impaired nerve function.

### Primary outcome

To assess the feasibility of neoadjuvant chemotherapy, we evaluated the surgical details, the postoperative complications, and the mortality rates. In the experimental group, this was done by an independent data monitoring committee that was not connected to the study. This comprised an oncology surgeon, a medical oncologist, and an independent statistician. For the historic cohort, all complications were reviewed by the independent team who performed the earlier study [21].

### Secondary outcomes

Secondary outcomes were blood loss, length of surgery, and body mass index at the time of diagnosis (as a surrogate marker of advanced disease status). For the control group, blood loss was only analyzed from 2010 because it was not recorded before that date.

#### Feasibility

Neoadjuvant chemotherapy was determined feasible if postoperative complications rates, perioperative blood loss, and length of surgery were comparable between the experimental and the historical control group.

### Analysis

Descriptive analyses of the treatments and outcomes were performed for patients in the experimental group. To assess differences between the experimental and control groups, 95% confidence intervals around the outcomes in the historical cohort were calculated, and we determined whether the values for the experimental patients were within these confidence limits.

## Results

**Fig. 1:**
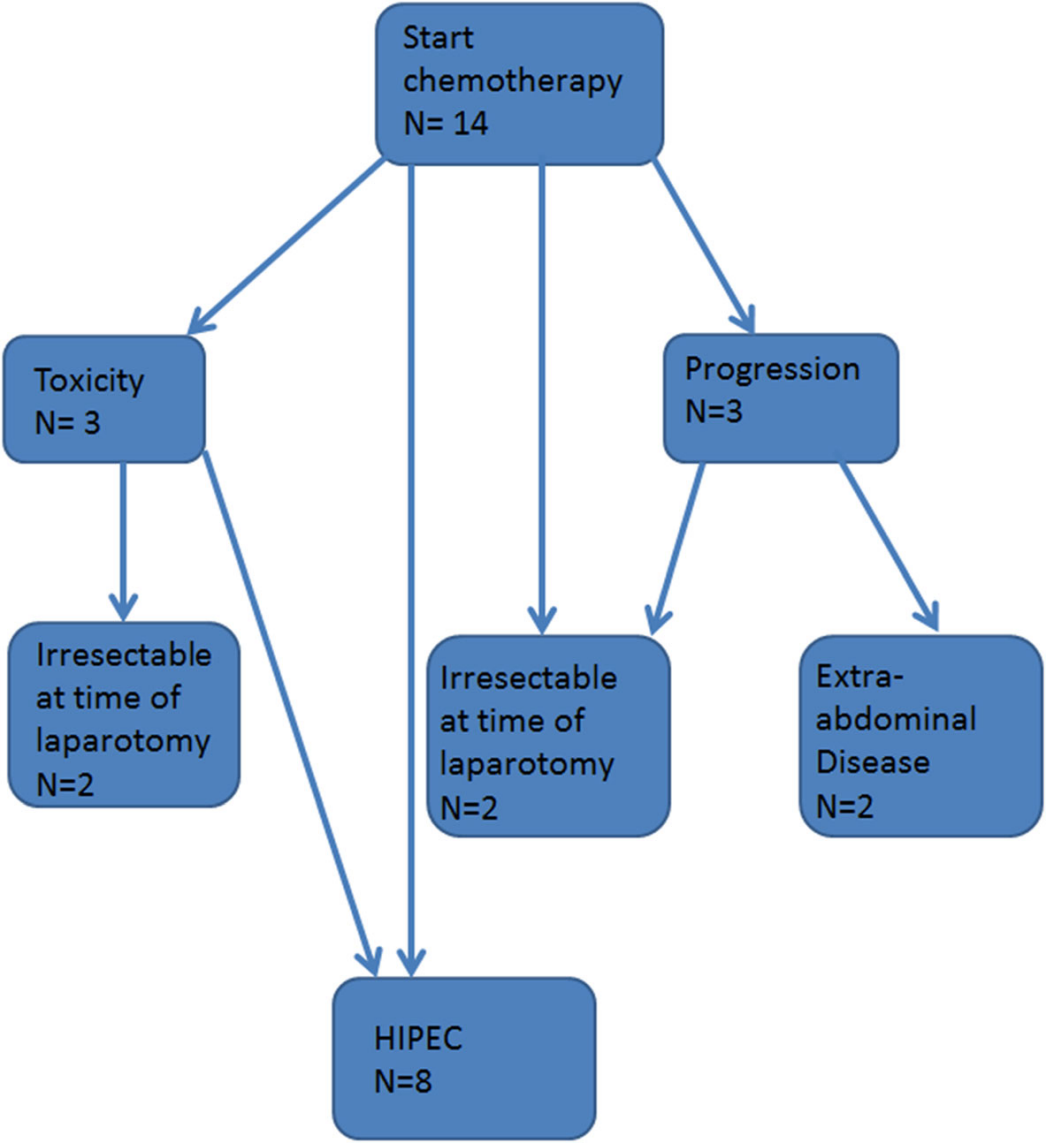
Flowchart of patients treatment

The baseline characteristics of the 14 patients in the experimental group and the 88 patients in the control group are summarized in Table 1. Overall, the experimental group was slightly older than the control group and had a slightly higher median body mass index. In the experimental group, peritoneal carcinomatosis was synchronous in 10 patients and metachronous in 4; in the control group, it was synchronous in 66 and metachronous in 22. Figure 1 illustrates the treatment of the patients in the experimental group.

**Table 1:** Patient and disease characteristics

	Experimental group *N* = 14	Control group *N* = 88
Age (median)	66.5 years (range 47–75 years)	59 years (range 26–76)
Gender	Female 42.9%	Female 51.8%
	Male 57.1%	Male 48.2%
BMI (median)	26.4 (range 20.31–35.08)	23.7 (range 11.47–40.04)
Origin of peritoneal carcinomatosis	Colorectal 14	Colorectal 110

*BMI* body mass index

### Neoadjuvant chemotherapy

An overview of the treatment and outcomes is provided for the experimental group in Table 2. Among the 14 patients in the experimental group, 4 required a dose reduction and 7 terminated neoadjuvant treatment early (disease progression in 3 patients and toxicity in 4 patients). Two of the patients with disease progression had extra-abdominal disease identified after one and four cycles of chemotherapy, respectively, and neither of these proceeded to CRS and HIPEC. Finally, one patient had a PCI > 20 after two cycles of chemotherapy (extensive intra-abdominal disease), one patient had a PCI > 20 after three cycles of chemotherapy, and one had a PCI > 20 after six cycles of chemotherapy, with all three being ineligible for CRS and HIPEC.

**Table 2:** Treatment and outcomes in the experimental group

Patient	Cycles of chemotherapy	Stopped	Reason for stopping	Extra-abdominal metastasis	Laparotomy	PCI	HIPEC	Current status
1	1	Yes	Toxicity	No	Yes	15	Yes, CC-0	DOD
2	6	No	–	No	Yes	26	No	DOD
3	6	No	–	No	Yes	7	Yes, CC-0	AWD
4	6	No	–	No	Yes	19	Yes, CC-0	DOD
5	3	Yes	Progression	No	Yes	35	No	DOD
6	6	No	–	No	Yes	9	Yes, CC-0	NED
7	6	No	–	No	Yes	1	Yes, CC-0	NED
8	6	No	–	No	Yes	4	Yes, CC-0	AWD
9	3	Yes	Progression	Yes	No	–	No	AWD
10	6	No	–	No	Yes	15	Yes, CC-0	NED
11	2	Yes	Toxicity	No	Yes	30	No	DOD
12	6	Yes	–	No	Yes	1	Yes, CC-0	NED
13	1	Yes	Progression	Yes	No	17	No	DOD
14	4	Yes	Toxicity	No	Yes	18	No	AWD

*DOD* death of disease, *AWD* alive with disease, *NED* no evidence of disease

### The CRS and HIPEC procedure

Ultimately, eight patients with a median PCI of 15.3 underwent CRS and HIPEC in the experimental group. In the control group, 88 patients underwent CRS and HIPEC before adjuvant chemotherapy. The details of these are compared in Table 3. As shown, median blood loss was broadly comparable between the two groups, but the lengths of surgery and hospital stay were both shorter in the experimental group.

**Table 3:** Comparison of CRS and HIPEC in the experimental and control groups

	Experimental group (*N* = 8)	Control group (*N* = 88)
Median duration of hospital stay	15 days (range 2–38)	15 days (range 4–63)
Median length of surgery (min)	485 (339–617)	502.50 (122–992)
Median blood loss (mL)	750 (100–2500)	700 (100–7000)
Resected organs		
Hemicolectomy	2/8	43/110
(Sub)total colectomy	0/8	10/110
Pelvic peritoneum	6/8	81/110
Splenectomy	3/8	31/110
Small bowel resection	4/8	26/110
Cholecystectomy	2/8	23/110

*CRS* cytoreductive surgery, *HIPEC* hyperthermic intraperitoneal chemotherapy

### Postoperative complications

The postoperative complications, and their rates, are summarized in Table 4. In the experimental group, gastrointestinal complications were most common, followed by pulmonary complications or sepsis, with cardiovascular or neurological complications being least common. In the control group, gastrointestinal problems were still most common, followed by nephrological and cardiovascular complications, which were at similar rates, and then pulmonary complications. Neurological complications and sepsis were least common in this group. Although there were no anastomotic leaks in the experimental group, one patient developed an enterocutaneous fistula; by contrast, 8% of the control group developed anastomotic leakage. There were no treatment-related deaths in either group.

**Table 4:** Complications after CRS and HIPEC

	Experimental group *N* = 8	Control group *N* = 88
Cardiovascular	1 (12.5%)	20 (18.1%)
Pulmonary	2 (25%)	12 (10.9%)
Gastrointestinal	3 (37.5%)	31 (28.2%)
Sepsis/SIRS	2 (25%)	9 (8.1%)
Mental	1 (12.5%)	12 (10.9%)
Wound	2 (25%)	7 (6.4%)
Anastomotic leakage	0 (0%)	8 (7.2%)
Neurological	1 (12.5%)	2 (1.8%)
Nephrological	0 (0%)	19 (17.3%)

*CRS* cytoreductive surgery, *HPEC* hyperthermic intraperitoneal chemotherapy

## Discussion

We investigated the feasibility and safety of neoadjuvant chemotherapy before CRS and HIPEC. Our results show that neoadjuvant chemotherapy did not influence either the morbidity or the mortality rate. However, half of the patients did need to stop neoadjuvant chemotherapy early due to disease progression or complications. In a study by Liu et al., it was reported that only 10.7% of patients needed to stop neoadjuvant therapy [26]. This difference may have arisen because the mean number of completed cycles in their study was 2.7, compared with 4.2 in ours. In a study in which FOLFOX (folinic acid, fluorouracil, and oxaliplatin) was used preoperatively, the researchers showed that only 14% had to terminate chemotherapy early [27]. This difference might be explained by the bigger sample size of the study and the fact that the goal of neo-adjuvant treatment was to make unresectable metastases resectable. If this was not possible, patients did not undergo surgery. However, patients in our study were required to be sufficiently healthy to tolerate CRS and HIPEC, so those with extensive complications were not included.

Three of our patients (21%) showed tumor progression during systemic chemotherapy, which is consistent with prior research experience. Indeed, studies of patients suffering from liver metastases due to CRC have shown that 11.2% (4/36) or 23% (3/13) had disease progression while receiving neoadjuvant chemotherapy [27, 28]. It can be argued that these patients may miss out on potentially curative surgery if they are selected for neoadjuvant treatment. However, failure of neoadjuvant treatment can be indicative of the fact that a tumor is either not chemosensitive or that the patient is too weak to undergo further treatment. As such, the additional value of CRS and HIPEC is questionable in these patients, and tumor progression during neoadjuvant treatment may be a contraindication to further surgery.

The rate of postoperative complications tends to be high after CRS and HIPEC, with reports giving rates ranging from 23 to 66% [2, 5, 12–15]. The most frequent complications are small bowel leakage, digestive fistulas, and abdominal sepsis [7, 13, 29]. Earlier studies of neoadjuvant chemotherapy in patients with colorectal liver metastases showed that there was a slight increase in postoperative infections and blood loss with the approach [16, 30]. In our study, however, the incidence of postoperative complications did not differ between the experimental and control groups. Compared with the historic control group, there were more pulmonary (25% vs 11.4%), sepsis (25% vs 8%), and neurological (12.5% vs 1.8%) complications in the experimental group, and there were fewer cardiovascular complications (12.5% vs 27.3%) and no renal complications (0% vs 19.3%).

To the best of our knowledge, this study is the first prospective study of the feasibility and safety of neoadjuvant chemotherapy before CRS and HIPEC. However, there are some important limitations to this research. Notably, the registration of complications in the historic control group was controlled by a different research team, and as such, was beyond our control. Also, the study design and aim meant that we included few patients from only one center, limiting our ability to generalize the results. Furthermore, this study did not take into account an eventual role of tumor biology or the influence of RAS/RAF mutations on the efficacy of neoadjuvant chemotherapy. Earlier studies have shown that that the reaction to targeted chemotherapy and overall survival might be influenced by these mutations in patients with peritoneal carcinomatosis from colorectal cancer. This should be taken into account when focusing on the efficacy of neoadjuvant chemotherapy in future trials [31, 32]. Finally, the median age was higher in the experimental group, but this may have had limited relevance because we observed no differences in complication rates. In the future, a prospective randomized controlled trial could answer the question whether neoadjuvant chemotherapy prior to CRS and HIPEC is effective in preventing hematogenous and lymphogenous metastases.

## Conclusion

In conclusion, postoperative complication rates, perioperative blood loss, and length of surgery are not increased by giving neoadjuvant treatment before CRS and HIPEC. Therefore, it can be considered feasible and safe to perform further study on the effects of neoadjuvant chemotherapy before CRS and HIPEC. In the future, we aim to investigate the efficacy of neoadjuvant chemotherapy in a multicenter trial, specifically assessing not only its effect on survival but also whether responsiveness to it could serve as a selection criterion for CRS and HIPEC.

## Abbreviations

CRS: Cytoreductive surgery
CT: Computed tomography
HIPEC: Hyperthermic intraperitoneal chemotherapy
PCI: Peritoneal cancer index
RECIST: Response evaluation criteria in solid tumors
UMCG: University Medical Center of Groningen
WHO: World Health Organization
